# Supplementary material for: Linking climate warming and land conversion to species’ range changes across Great Britain
Source: Nat Commun. 2023 Oct 30;14:6759. doi: 10.1038/s41467-023-42475-0 (PMC10616271; doi:10.1038/s41467-023-42475-0)
Supplement: Supplementary file 1 — Supplementary Information [file 41467_2023_42475_MOESM1_ESM.pdf]

# Supplementary Information for

# Linking climate warming and land conversion to species' range changes across Great Britain

Andrew J. Suggitt, Christopher J. Wheatley, Paula Aucott, Colin M. Beale, Richard Fox, Jane K. Hill, Nick J. B. Isaac, Blaise Martay, Humphrey Southall, Chris D. Thomas, Kevin J. Walker & Alistair G. Auffret.

Correspondence to: andrew.suggitt@northumbria.ac.uk, alistair.auffret@slu.se.

**This file includes:**

Figure S1- Tree diagram of the relative frequency of 'best' models for each species.

Figure S2- Comparison of distribution change metrics using the Frescalo and Telfer methods.

Figure S3- Tree diagram of the relative frequency of 'best' models for each species (habitat specialists only).

Figure S4. Average Marginal Effects (AMEs) of a 10% change in habitat (habitat specialists only).

Table S1- Matching scheme for land classes.

Table S2- Table of time windows for each dataset (historical and modern).

Table S3- Variables included in the model sets for taxon-level and species-level statistical modelling.

Table S4- Taxon-level model output tables.

Table S5- Taxon-level linear mixed model R-squared values.

Table S6- Species-level comparison of predicted persistence values generated by models with and without an interaction term (interactive vs additive comparison).

Table S7- Translation of grid-square persistence into distribution trends across Great Britain.

Supplementary Data 1- Persistence rates for each study species (separate file).

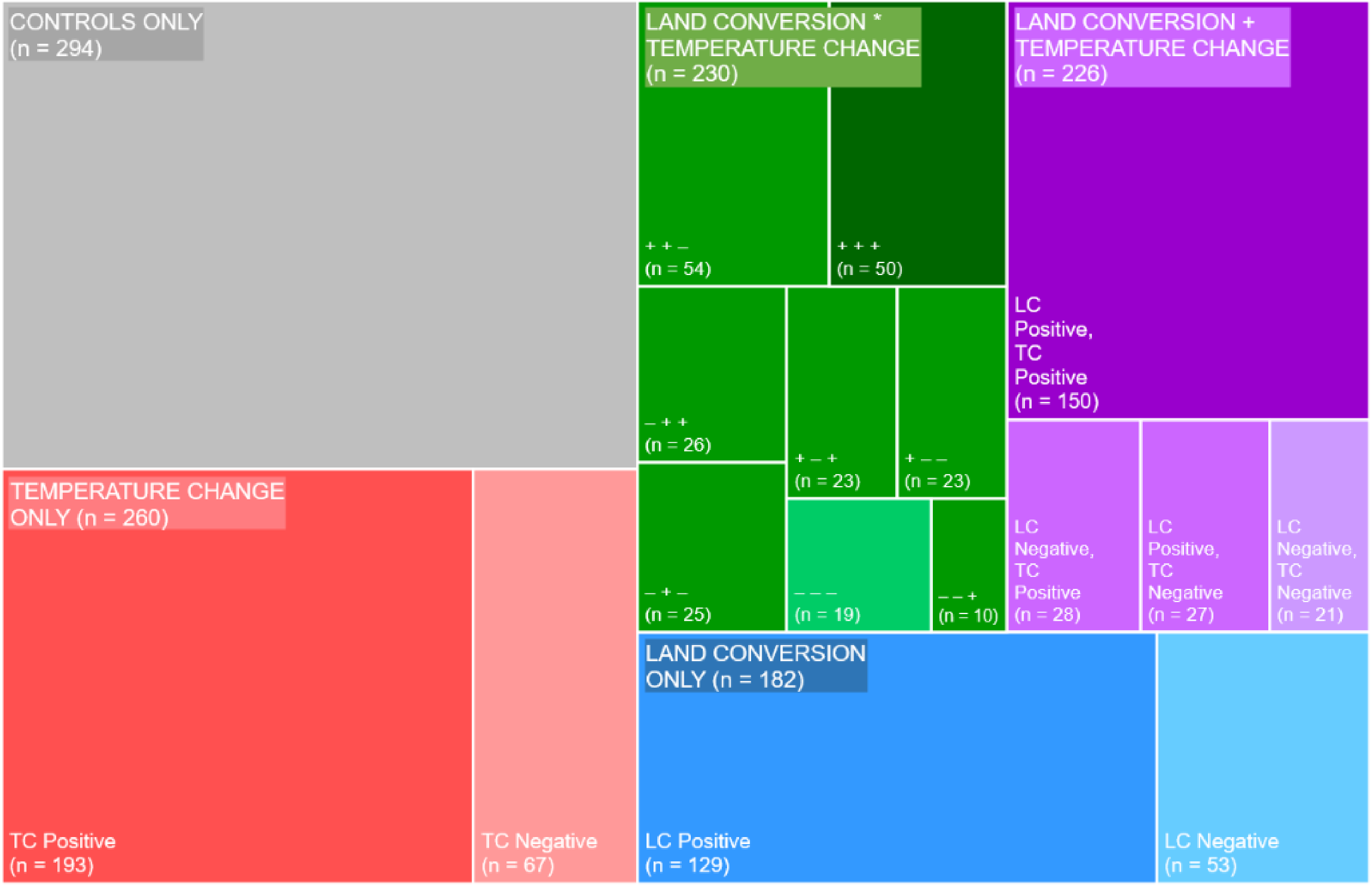


**Fig. S1.** Tree diagram of the relative frequency of 'best' statistical models for each species, and the responses to environmental change they identified. The size of each rectangle is proportional to the number of species in each category of model and their response (positive or negative), whilst colours indicate which one of the five overall model formulations was 'best' (see 'Statistical Modelling' section for formulations).

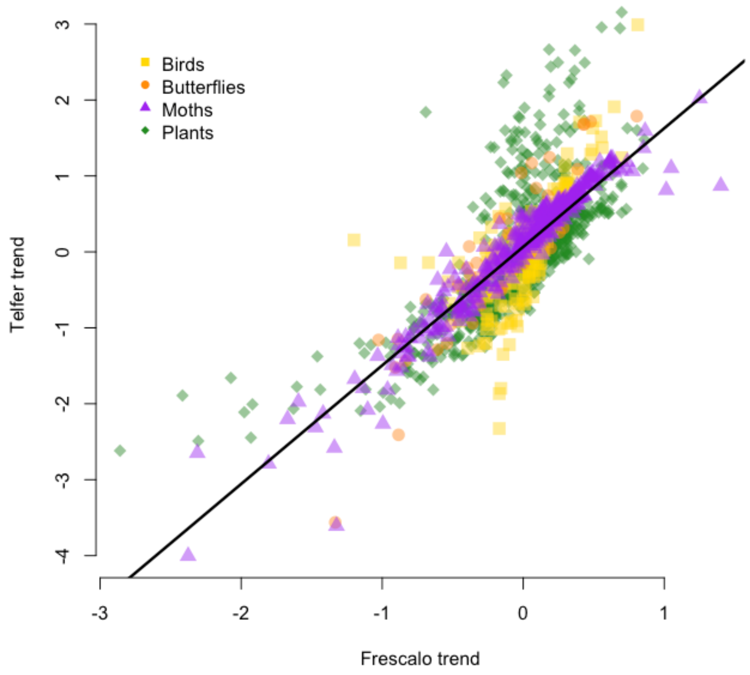


**Fig. S2. Comparison of distribution change metrics using the Frescalo and Telfer methods.** Line is the result of a linear model regressing the methods against each other, including all species of all taxon. Model R-squared is 0.63.

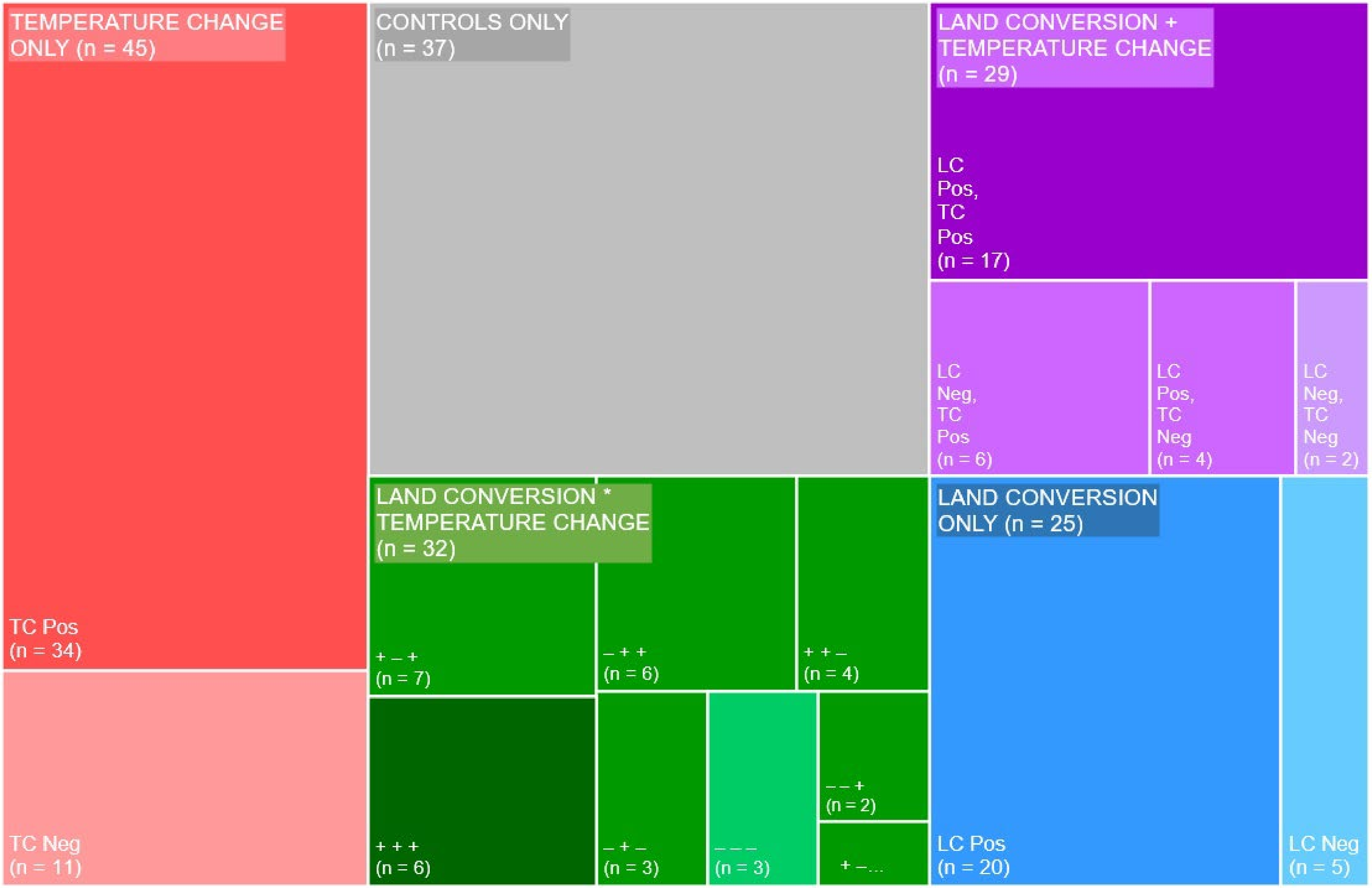


**Fig. S3. Tree diagram of the relative frequency of 'best' statistical models for each specialist species, and the responses to environmental change they identified.** The size of each rectangle is proportional to the number of species in each category of model and their response (positive or negative), whilst colours indicate which one of the five overall model formulations was 'best' (see 'Statistical Modelling' section for formulations).

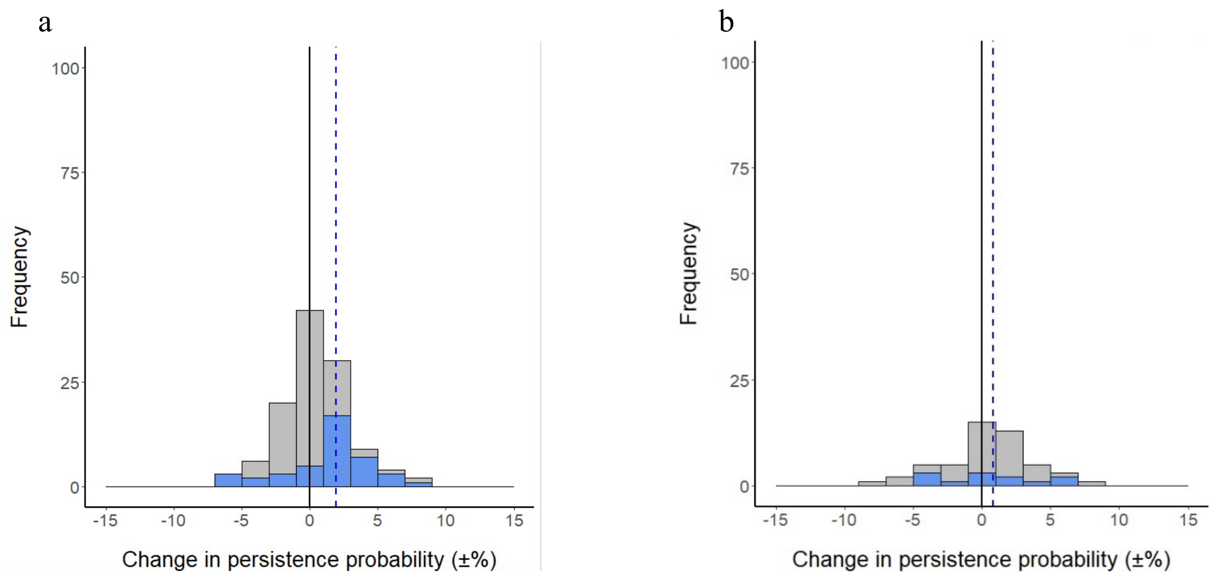


**Fig. S4. Average Marginal Effects (AMEs) of a 10% change in habitat for (a) grassland specialist species (n = 116) and (b) woodland specialist species (n = 52).** All 168 species are illustrated in grey, with coloured bars representing the subsets of species for which the respective variable was included in the 'best' model (excluding species exhibiting interactive responses, n = 32).

**Table S1. Scheme for matching LUSGB land use types to CEH land cover classes.** Classes from the CEH Land Cover Map (LCM) 2007 appear in the left-hand columns, with the Dudley Stamp Land Use Survey (LUSGB) classes they were mapped onto in the third column. There were some cases where the delineation between particular classes was unclear, particularly around the coastline, and so some were either removed or amalgamated (fourth column).

| CEH LCM 2007 classes (n = 23) | CEH ID in 25m raster | LUSGB 1933-49 land use classes (n = 8) | Our broad land use categories (n = 6) |
|---|---|---|---|
| Urban | 22 | Urban | Urban |
| Suburban | 23 | Suburban | |
| Broadleaved woodland | 1 | Woodland | Woodland |
| Coniferous woodland | 2 | | |
| Arable and horticulture | 3 | Arable | Arable |
| Improved grassland | 4 | Not mapped | Agriculturally-improved grassland |
| Neutral grassland | 6 | Grassland | Grassland |
| Calcareous grassland | 7 | | |
| Fen, marsh and swamp | 9 | | |
| Rough grassland | 5 | Rough grazing | |
| Acid grassland | 8 | | |
| Heather | 10 | | |
| Heather grassland | 11 | | |
| Bog | 12 | | |
| Montane habitats | 13 | | |
| Inland rock | 14 | | |
| Saltmarsh | 21 | Delineation unclear (Removed) | (Removed) |
| Saltwater | 15 | | |
| Freshwater | 16 | Water | Water |
| Littoral rock | 19 | Unpredictably assigned to the above (Removed) | (Removed) |
| Littoral sediment | 20 | | |
| Supra-littoral rock | 17 | | |
| Supra-littoral sediment | 18 | | |

**Table S2. Time windows of record coverage for each taxon.** The time windows for each taxon were selected to provide comparable spatial coverage of Great Britain in both the historical and the modern time periods of this study. Time windows tend to be larger in the historical period because recording effort was lower at this time (see 'n unique species-hectad records' entires). Recording effort- quantified here as the number of unique species-hectad records- increased over time for all four taxa.

<table>
<tr><th colspan="2">Data type</th><th>Historical period</th><th>Modern period</th></tr>
<tr><td colspan="2">Land conversion data</td><td>1933-49<br>Source: Dudley Stamp Land Use Survey (Stamp 1931).</td><td>2005-09<br>Source: Centre of Ecology Land Cover Map 2007 (Morton et al. 2011).</td></tr>
<tr><td rowspan="4">Distribution data</td><td>Birds</td><td>1968-72<br>Source: Sharrock atlas recording period (Sharrock et al. 1976).<br>n unique species-hectad records: 217,351.</td><td>2007-11<br>Source: Balmer atlas recording period (Balmer et al. 2013).<br>n unique species-hectad records: 313,302.</td></tr>
<tr><td>Butterflies</td><td rowspan="2">1930-60<br>Source: Butterflies of the New Millennium and National Moth Recording Scheme datasets (Butterfly Conservation). Note that the historical period precedes butterfly atlas coverage by 10-20 years, and macromoth atlas coverage by 40+ years.<br>n unique species-hectad records (butterflies): 20,611.<br>n unique species-hectad records (moths): 79,324.</td><td rowspan="2">2005-09<br>Source: Five year atlas recording period (Butterfly Conservation 2020).<br>n unique species-hectad records (butterflies): 51,539<br>n unique species-hectad records (moths): 355,429.</td></tr>
<tr><td>Macromoths</td></tr>
<tr><td>Plants</td><td>1930-60<br>Source: Perring & Walters atlas recording period (Perring & Walters 1962).<br>n unique species-hectad records: 273,609.</td><td>2000-15<br>Source: BSBI Atlas 2020 recording period (BSBI 2020).<br>n unique species-hectad records: 967,467.</td></tr>
<tr><td colspan="2">Climate warming data</td><td colspan="2">Warming for each grid square was calculated over each taxon's interim period, i.e. the climate warming grid for plants was calculated on data for 1961-99, birds for 1973-2006, and Lepidoptera (butterflies and macromoths) 1961-2004.<br>Source: UKCP18 Observed climate data (UKMO 2020).</td></tr>
</table>

**Table S3. Variables included in the model sets for taxon-level and species-level statistical modelling.**

| Model number | Model name | Model formula | Definitions |
|---|---|---|---|
| #1 | Controls only | Persistence/Extinction ~ Microclimatic buffering + Recorder effort + PCNM1 + PCNM2 | Microclimatic buffering: Potential for variation in microclimate arising from topography.<br><br>Recorder effort: Sampling effort for each taxon in the neighbourhood of each grid square, derived from Frescalo.<br><br>PCNM 1 & 2: Spatial autocorrelation control. The first two eigenvectors of a principal coordinates analysis derived from a neighbour matrix of the grid square centroids. |
| #2 | Climate warming only | (As per #1) + Climate warming | Climate warming: Rate of change in temperature for the target grid square (°C / decade), measured over the relevant atlas period. |
| #3 | Land conversion only | (As per #1) + Land conversion | Land conversion: The proportion of pixels within each grid cell that changed land-use classes between time-periods |
| #4 | Additive model (Climate warming + land conversion) | (As per #1) + Climate warming + Land conversion | (no new model terms) |
| #5 | Interactive model (Climate warming * land conversion) | (As per #1) + Climate warming * Land conversion | (no new model terms) |

**Table S4. Best performing taxon-level Linear Mixed Model (LMM) output tables.** Each taxon was modelled separately with species identity fitted as a random effect: **A** birds (n = 141 species), **B** butterflies (n = 47 species), **C** macromoths (n = 333 species) and **D** plants (n = 680 species).

**A** BIRDS

| **Term** | **Estimate** | **Lower CI (2.5%)** | **Upper CI (97.5%)** | **z-value** | **P-value** | **Sig** |
|---|---|---|---|---|---|---|
| Intercept | 0.76 | 0.43 | 1.21 | 3.57 | 0.0004 | *** |
| Land conversion | 1.49 | 1.12 | 1.81 | 6.35 | < 0.0001 | *** |
| Temperature change | 4.23 | 3.28 | 4.80 | 10.44 | < 0.0001 | *** |
| Recorder effort | 0.08 | 0.0003 | 0.17 | 1.79 | 0.0743 | |
| Microclimatic buffering | -10.98 | -13.21 | -9.20 | -8.54 | < 0.0001 | *** |
| PCNM 1 | 5.04 | 3.60 | 6.42 | 7.28 | < 0.0001 | *** |
| PCNM 2 | 2.00 | 1.35 | 2.85 | 4.46 | < 0.0001 | *** |
| Land conversion: temperature change | -3.78 | -4.64 | -2.71 | -5.91 | < 0.0001 | *** |

**B** BUTTERFLIES

| **Term** | **Estimate** | **Lower CI (2.5%)** | **Upper CI (97.5%)** | **z-value** | **P-value** | **Sig** |
|---|---|---|---|---|---|---|
| Intercept | -1.32 | -2.09 | -0.49 | -3.27 | 0.0011 | ** |
| Land conversion | 0.68 | 0.40 | 0.94 | 4.51 | < 0.0001 | *** |
| Temperature change | 5.54 | 4.21 | 6.76 | 7.07 | < 0.0001 | *** |
| Recorder effort | 0.89 | 0.72 | 1.04 | 9.22 | < 0.0001 | *** |
| Microclimatic buffering | 9.72 | 3.25 | 18.65 | 2.51 | 0.01 | * |
| PCNM 1 | 4.61 | 2.08 | 6.29 | 4.58 | < 0.0001 | *** |
| PCNM 2 | 8.55 | 7.13 | 9.74 | 10.78 | < 0.0001 | *** |

**C** MACROMOTHS

| **Term** | **Estimate** | **Lower CI (2.5%)** | **Upper CI (97.5%)** | **z-value** | **P-value** | **Sig** |
|---|---|---|---|---|---|---|
| Intercept | -1.20 | -1.41 | -0.93 | -8.84 | < 0.0001 | *** |
| Land conversion | 0.24 | 0.11 | 0.34 | 3.94 | < 0.0001 | *** |
| Temperature change | 4.34 | 3.42 | 4.87 | 12.43 | < 0.0001 | *** |

| | | | | | | |
|---|---|---|---|---|---|---|
| Recorder effort | 0.68 | 0.61 | 0.77 | 17.30 | < 0.0001 | *** |
| Microclimatic buffering | -16.42 | -20.03 | -13.91 | -9.85 | < 0.0001 | *** |
| PCNM 1 | -5.32 | -6.13 | -4.74 | -14.62 | < 0.0001 | *** |
| PCNM 2 | 3.21 | 2.31 | 3.99 | 7.28 | < 0.0001 | *** |

**D** PLANTS

| **Term** | **Estimate** | **Lower CI (2.5%)** | **Upper CI (97.5%)** | **z-value** | **P-value** | **Sig** |
|---|---|---|---|---|---|---|
| Intercept | 0.81 | 0.65 | 0.94 | 11.45 | < 0.0001 | *** |
| Land conversion | 0.33 | 0.26 | 0.38 | 9.55 | < 0.0001 | *** |
| Temperature change | 2.12 | 1.75 | 2.59 | 10.70 | < 0.0001 | *** |
| Recorder effort | 0.19 | 0.14 | 0.23 | 7.98 | < 0.0001 | *** |
| Microclimatic buffering | 3.72 | 2.41 | 5.19 | 5.15 | < 0.0001 | *** |
| PCNM 1 | 8.69 | 7.75 | 9.46 | 17.25 | < 0.0001 | *** |
| PCNM 2 | -1.13 | -1.86 | -0.56 | -3.36 | 0.0008 | *** |

**Table S5. The R-squared values for the best performing Linear Mixed Model (LMM) for each taxon.** Models were formulated as per Table S2 above. Conditional R-squared values represent the proportion of variation explained by the random effect, in this case species identity. Marginal R-squared values represent the proportion of variation explained by the fixed effects- environmental change variables plus controls.

| Taxon | R-squared | | | |
|---|---|---|---|---|
| | Conditional | | Marginal | |
| | Theoretical | Delta | Theoretical | Delta |
| Birds | 0.47 | 0.30 | 0.02 | 0.01 |
| Butterflies | 0.57 | 0.48 | 0.04 | 0.03 |
| Macromoths | 0.25 | 0.21 | 0.05 | 0.04 |
| Plants | 0.31 | 0.20 | 0.02 | 0.01 |

**Table S6. The size and frequency of differences in predicted probability of persistence (±%) between models fitted with and without land use change * climate change interaction terms for the 230 species where interaction models were identified as the 'best' model.** Persistence probability was predicted for each species by substituting the minimum, maximum and the intervening decile (10th-90th percentile) observed values for land conversion (n = 11 observed values) and for temperature change (n = 11) into model equations from the 'best' model for each species (n = 11 x 11 = 121 predictions of persistence probability for each interactive model), and equivalent predictions were generated from (otherwise identical) models omitting the interaction term (n = 121 predictions of persistence probability for each additive model). Differences in the predicted values between the two model formulations were calculated by subtracting the 90$^{th}$ percentile of the predicted values from the interactive model from the 90$^{th}$ percentile of the predicted values from the additive model (top row), and so on for each decile. Columns indicate the total number of species with predicted differences of each magnitude (within ±10% of each other, differ by ±10 – 20%, etc) at each decile of the comparison.

| Percentile of comparison between interactive and additive models (persistence probability, %) | Number of species for which predicted values for interactive models and additive models… | | | | | |
|---|---|---|---|---|---|---|
| | Were within ±10% | Differed by 10 to 20% | Differed by 20 to 30% | Differed by 30 – 40% | Differed by 40 – 50% | Differed by > 50% |
| | ● | ● | ● | ● | ● | ● |
| 90$^{th}$ (extremely high) | 18 | 68 | 67 | 35 | 18 | 24 |
| 80$^{th}$ (very high) | 75 | 87 | 45 | 8 | 10 | 5 |
| 70$^{th}$ (high) | 127 | 74 | 17 | 9 | 2 | 1 |
| 60$^{th}$ (medium to high) | 161 | 58 | 9 | 2 | 0 | 0 |
| 50$^{th}$ (medium) | 197 | 29 | 4 | 0 | 0 | 0 |
| 40$^{th}$ (low to medium) | 214 | 15 | 1 | 0 | 0 | 0 |
| 30$^{th}$ (low) | 229 | 1 | 0 | 0 | 0 | 0 |
| 20$^{th}$ (very low) | 229 | 1 | 0 | 0 | 0 | 0 |
| 10$^{th}$ (extremely low) | 230 | 0 | 0 | 0 | 0 | 0 |

**Table S7. Translation of grid-square persistence into distribution trends across Great Britain.** Numbers of species are sorted into columns based on the identity of their 'best' model and whether national-level trends calculated by the Frescalo algorithm indicated positive or negative trends (n = 668 species for which single-effect or additive models were deemed the 'best' model), with total numbers of species provided below. Values are provided for all species together (equivalent to Fig. 3) and separately by taxa.

| | **Land conversion** | | **Temperature change** | | **Land conversion + Temperature change** | | | | |
|---|---|---|---|---|---|---|---|---|---|
| | LC + | LC - | TC+ | TC- | LC+ TC+ | LC+ TC- | LC- TC+ | LC- TC- | **Total** |
| **All taxa** | | | | | | | | | |
| Positive trend | 18 | 75 | 23 | 119 | 102 | 9 | 13 | 3 | 362 |
| Negative trend | 34 | 54 | 44 | 74 | 48 | 18 | 15 | 17 | 304 |
| **Total** | 53 | 129 | 67 | 193 | 150 | 27 | 28 | 21 | 668 |
| **Plants** | | | | | | | | | |
| Positive trend | 7 | 48 | 17 | 72 | 71 | 7 | 6 | 2 | 230 |
| Negative trend | 25 | 29 | 29 | 41 | 20 | 15 | 7 | 14 | 180 |
| **Total** | 33 | 77 | 46 | 113 | 91 | 22 | 13 | 17 | 412 |
| **Birds** | | | | | | | | | |
| Positive trend | 4 | 12 | 2 | 3 | 13 | 1 | 2 | 0 | 37 |
| Negative trend | 3 | 14 | 2 | 5 | 14 | 1 | 2 | 0 | 41 |
| **Total** | 7 | 26 | 4 | 8 | 27 | 2 | 4 | 0 | 78 |
| **Macromoths** | | | | | | | | | |
| Positive trend | 7 | 14 | 4 | 40 | 13 | 1 | 4 | 1 | 84 |
| Negative trend | 6 | 11 | 9 | 24 | 7 | 1 | 4 | 1 | 63 |
| **Total** | 13 | 25 | 13 | 64 | 20 | 2 | 8 | 2 | 147 |
| **Butterflies** | | | | | | | | | |
| Positive trend | 0 | 1 | 0 | 4 | 5 | 0 | 1 | 0 | 11 |
| Negative trend | 0 | 0 | 4 | 4 | 7 | 1 | 2 | 2 | 20 |
| **Total** | 0 | 1 | 4 | 8 | 12 | 1 | 3 | 2 | 31 |
