# Supplementary material for: Linking climate warming and land conversion to species’ range changes across Great Britain
Source: Nat Commun. 2023 Oct 30;14:6759. doi: 10.1038/s41467-023-42475-0 (PMC10616271; doi:10.1038/s41467-023-42475-0)
Supplement: Supplementary file 4 — Reporting Summary [file 41467_2023_42475_MOESM4_ESM.pdf]

nature portfolio

Corresponding author(s): Andrew Suggitt

Last updated by author(s): Aug 7, 2023

Reporting Summary

Nature Portfolio wishes to improve the reproducibility of the work that we publish. This form provides structure for consistency and transparency in reporting. For further information on Nature Portfolio policies, see our Editorial Policies and the Editorial Policy Checklist.

Statistics

For all statistical analyses, confirm that the following items are present in the figure legend, table legend, main text, or Methods section.

n/a	Confirmed	
☐	☒	The exact sample size (*n*) for each experimental group/condition, given as a discrete number and unit of measurement
☒	☐	A statement on whether measurements were taken from distinct samples or whether the same sample was measured repeatedly
☐	☒	The statistical test(s) used AND whether they are one- or two-sided<br>*Only common tests should be described solely by name; describe more complex techniques in the Methods section.*
☐	☒	A description of all covariates tested
☐	☒	A description of any assumptions or corrections, such as tests of normality and adjustment for multiple comparisons
☐	☒	A full description of the statistical parameters including central tendency (e.g. means) or other basic estimates (e.g. regression coefficient) AND variation (e.g. standard deviation) or associated estimates of uncertainty (e.g. confidence intervals)
☐	☒	For null hypothesis testing, the test statistic (e.g. *F*, *t*, *r*) with confidence intervals, effect sizes, degrees of freedom and *P* value noted<br>*Give P values as exact values whenever suitable.*
☒	☐	For Bayesian analysis, information on the choice of priors and Markov chain Monte Carlo settings
☐	☒	For hierarchical and complex designs, identification of the appropriate level for tests and full reporting of outcomes
☐	☒	Estimates of effect sizes (e.g. Cohen's *d*, Pearson's *r*), indicating how they were calculated

Our web collection on statistics for biologists contains articles on many of the points above.

Software and code

Policy information about availability of computer code

Data collection	A large amount of primary and secondary data were generated and analysed for the preparation of this manuscript. Full details of these data, and how they can be accessed online by the public, are available in the methods section and throughout the text of the manuscript itself.
Data analysis	Code is available on figshare at 10.6084/m9.figshare.23925462. Analyses were performed in R software (v 4.1.0). For a fully-referenced description of the R packages we used (inc. version numbers), please see the methods section of the manuscript.

For manuscripts utilizing custom algorithms or software that are central to the research but not yet described in published literature, software must be made available to editors and reviewers. We strongly encourage code deposition in a community repository (e.g. GitHub). See the Nature Portfolio guidelines for submitting code & software for further information.

Data

Policy information about availability of data

All manuscripts must include a data availability statement. This statement should provide the following information, where applicable:

- Accession codes, unique identifiers, or web links for publicly available datasets
- A description of any restrictions on data availability
- For clinical datasets or third party data, please ensure that the statement adheres to our policy

The land use data generated and analysed in this study have been deposited online at https://doi.org/10.5878/9wks-qg91. The full resolution land-use data remain

April 2023

under copyright and are not available due to data privacy laws. The climate data used in this study are available on the UKCP18 database at https://ukclimateprojections-ui.metoffice.gov.uk. The biodiversity data used in this study are available on the NBN Gateway at https://nbnatlas.org.

Research involving human participants, their data, or biological material

Policy information about studies with human participants or human data. See also policy information about sex, gender (identity/presentation), and sexual orientation and race, ethnicity and racism.

Reporting on sex and gender	N/A
Reporting on race, ethnicity, or other socially relevant groupings	N/A
Population characteristics	N/A
Recruitment	N/A
Ethics oversight	N/A

Note that full information on the approval of the study protocol must also be provided in the manuscript.

Field-specific reporting

Please select the one below that is the best fit for your research. If you are not sure, read the appropriate sections before making your selection.

☐ Life sciences ☐ Behavioural & social sciences ☒ Ecological, evolutionary & environmental sciences

For a reference copy of the document with all sections, see nature.com/documents/nr-reporting-summary-flat.pdf

Ecological, evolutionary & environmental sciences study design

All studies must disclose on these points even when the disclosure is negative.

Study description	A multispecies study of the interactive effects of land-use and climate change on the biota. The distribution change of each species effectively acted as a replicate, although species were modelled on a species-by-species basis. Models were constructed in the basic form extirpation/persistence (0/1) ~ warming + land conversion + warming*land conversion.
Research sample	Sample included all species in the study region with sufficient data (there was no sampling strategy).
Sampling strategy	Monitoring strategies for each taxon dataset (national recording schemes) are set out in the associated publications that support them. The logic of these strategies is set out in the front matter of publications 10-12 in the manuscript reference list.
Data collection	Our study is the product of over 22 million citizen science records, which themselves are the product of a number of campaigns to collect species' distribution data. Full details of how these data were assembled are available in the above references (see sampling strategy).
Timing and spatial scale	Biodiversity data were collected from 1930 until 2015, with climate data collected and analysed to suit periods of biodiversity data recording. The spatial extent of the study is the island of Great Britain and the spatial (grid square) resolution is 10 x 10 km.
Data exclusions	Some 26 species (of a full dataset of 1,218 species) were excluded for data quality purposes. Full details of the inclusion criteria are set out in the Methods.
Reproducibility	Several runs of statistical models under various assumptions and frameworks were made before alighting on the final workflow for model selection and species classification. This included three variant modelling approaches (with spatial-autocorrelation control, without spatial autocorrelation control, and with an alternative spatial correlation control generated via the geeglm:geese), all of which yielded similar results, so for simplicity we opted for the approach we describe in the manuscript and methods.
Randomization	Randomisation not relevant here as our study is observational.
Blinding	Blinding is not relevant here as our study is observational.

Did the study involve field work? ☐ Yes ☒ No

Reporting for specific materials, systems and methods

We require information from authors about some types of materials, experimental systems and methods used in many studies. Here, indicate whether each material, system or method listed is relevant to your study. If you are not sure if a list item applies to your research, read the appropriate section before selecting a response.

Materials & experimental systems

n/a	Involved in the study
☒	☐ Antibodies
☒	☐ Eukaryotic cell lines
☒	☐ Palaeontology and archaeology
☒	☐ Animals and other organisms
☒	☐ Clinical data
☒	☐ Dual use research of concern
☒	☐ Plants

Methods

n/a	Involved in the study
☒	☐ ChIP-seq
☒	☐ Flow cytometry
☒	☐ MRI-based neuroimaging
